# Role of parental characteristics and feeding practices in childhood obesity: a cross-sectional study

**DOI:** 10.1136/bmjpo-2026-004819

**Published:** 2026-06-04

**Authors:** Seda Önal, Hilal Şimsek, Aslı Uçar

**Affiliations:** 1 Faculty of Health Sciences, Department of Nutrition and Dietetics, Firat Universitesi, Elâzığ, Turkey; 2 Bor Faculty of Health Sciences, Department of Nutrition and Dietetics, Nigde Omer Halisdemir Universitesi, Nigde, Turkey; 3 Faculty of Health Sciences, Department of Nutrition and Dietetics, Ankara Universitesi, Ankara, Turkey

**Keywords:** Obesity, Child

## Abstract

**Background:**

Childhood obesity represents a growing global public health challenge, and parental characteristics together with family feeding practices are increasingly recognised as important determinants of children’s dietary behaviours and weight status. The present study aimed to investigate the associations between parental characteristics, feeding practices and the risk of obesity among school-aged children.

**Methods:**

A total of 446 parent–child dyads, consisting of primary school–aged children and one of their parents, were included in the study. In this cross-sectional study, parental feeding practices and the family environment were evaluated using the Child Feeding Questionnaire and the Family Nutrition and Physical Activity tool. Factors associated with age-standardised child body mass index (BMI) were identified using multivariable linear regression, with restricted cubic splines employed to explore potential non-linear associations with parental BMI.

**Results:**

The prevalence of obesity was 18.2% among the children. Factors positively associated with child BMI were maternal BMI (β=0.136), perceived child weight (β=0.143) and concern about child weight (β=0.133), while pressure to eat was negatively associated (β=−0.218; all p<0.01). In adjusted models, parental BMIs showed non-linear associations (p_nonlinearity_<0.05), levelling off for mothers while intensifying for fathers at higher BMI levels.

**Conclusions:**

Parental characteristics—particularly maternal BMI, educational level and feeding practices—play a significant role in the development of childhood obesity. Family-centred preventive strategies addressing parental behaviours and awareness may contribute to more effective obesity prevention and support the development of public health policies.

**WHAT IS ALREADY KNOWN ON THIS TOPIC**

- Characterised by its multifactorial aetiology, childhood obesity is a global public health concern influenced by environmental and behavioural factors, including parental feeding practices.

**WHAT THIS STUDY ADDS**

- This study highlights that the integration of parental sociodemographic characteristics and specific feeding behaviours explains childhood obesity risk more comprehensively than isolated assessments.

**HOW THIS STUDY MIGHT AFFECT RESEARCH, PRACTICE OR POLICY**

- These findings could contribute to the development of targeted, family-centred interventions and public health policies that prioritise parental education and adaptive, non-restrictive feeding practices.

## Introduction

 Childhood obesity represents a major public health concern in the 21st century.^1 2^ As of 2022, the global prevalence of overweight among children and adolescents aged 5–19 reached over 390 million, with 160 million categorised as children with obesity.^3^ In addition, it is estimated that 206 million children and youth between the ages of 5–19 will face obesity in 2025 and 254 million in 2030.^4^ Childhood obesity is particularly concerning due to its immediate health consequences and long-term impact on well-being. Increased adiposity levels are closely linked to the development of metabolic disorders and early markers of cardiometabolic disease.^5^ Children with obesity have a significantly increased likelihood of remaining at risk of obesity in adulthood.^6 7^ A 2020 analysis of 12 142 individuals in the International Childhood Cardiovascular Cohort Consortium revealed that 56% of children with obesity and 80% of those with severe obesity developed obesity in adulthood.^8^

It is well established that obesity results from an energy imbalance, with a sustained positive energy balance significantly influenced by lifestyle behaviours and nutritional choices.^2^ Multiple factors also contribute to the onset of childhood obesity, including genetic predisposition, environmental conditions, eating behaviours, lack of physical activity and socioeconomic background.^9^ A study reported that factors such as parents’ perception of their child’s body weight, the child’s age and sex, rapid eating habits, a family history of obesity and parental neglect were linked to childhood obesity.^10^ Although these factors play a role in the risk of obesity, the family environment can further intensify these vulnerabilities. Even with good intentions, families may inadvertently foster an obesogenic environment—characterised by poor dietary habits and insufficient physical activity—that heightens the risk of children becoming overweight.^11^

Beyond contributing to an obesogenic environment, controlling feeding practices by parents may negatively influence children’s food preferences and disrupt their ability to self-regulate food intake—such as eating when not hungry or failing to respond to satiety cues—thereby increasing the risk of overweight or obesity.^12^ Despite the well-documented detrimental impacts of parental feeding practices, the specific interplay between these behaviours and distinct family environment factors remains insufficiently explored. Moreover, previous studies have largely examined parental feeding practices, parental body mass index (BMI) and family environment in isolation, leaving their relative contributions and potential non-linear associations with child BMI inadequately understood. However, these factors are inherently interrelated and may jointly influence child BMI through complex and potentially non-linear pathways. Addressing this gap is crucial, as a more comprehensive understanding of these interconnected factors may inform the development of more effective, targeted prevention programmes. Consequently, this study aimed to investigate the combined influence of family environment, parental feeding practices and parental anthropometric characteristics on the development of childhood obesity.

## Materials and methods

### Sample of the study

The sample of this cross-sectional study consisted of school-age children attending primary school in Ankara, Türkiye and their parents. A convenience sampling technique was employed; child–mother–father triads were invited from state primary schools, and face-to-face interviews were conducted with those who volunteered to participate. The inclusion criteria were children aged 8–11 years, current enrolment in primary school, and the mutual consent of all three parties (child, mother and father). Only triads in which complete data were obtained from all participants were included in the final analysis. Children with chronic illnesses, developmental disabilities, or medical conditions affecting growth, nutrition, or eating behaviours were excluded.

A total of 457 triads were initially recruited, and the final analysis was conducted with 446 triads after excluding those with incomplete data. The required sample size was determined using G*Power V.3.1 software. Based on a multiple linear regression model (f^2^: 0.08, α: 0.05, 1−β: 0.95) incorporating 13 predictors, a minimum of 344 participants was required, ensuring the final sample was sufficiently powered for the intended analyses. Patients or the public were not involved in the design, or conduct, or reporting, or dissemination plans of our research.

### Data collection

In this research, data collection was carried out using a face-to-face interview technique. The survey form includes the following data: demographic characteristics of parents and children, anthropometric measurements, Child Feeding Questionnaire (CFQ) and Family Nutrition and Physical Activity (FNPA) scale.

The body composition analysis and height measurements were taken by trained researchers according to standard procedures.^13^ Body composition was analysed by BIA (Tanita SC330), height was measured with a stadiometer fixed to the wall (SECA). BMI was calculated by dividing body weight (kg) by the square of height (m^2^). BMI-for-age percentiles for children were calculated using the WHO AnthroPlus software (V.1.0.4), based on WHO growth standards. Underweight was defined as <5th percentile, healthy weight was defined as ≥5th to <85th percentile, overweight was defined as ≥85th to <95th percentile and obesity was defined as ≥95th percentile of BMI-for-age.^14^

The FNPA scale, developed to evaluate the obesogenic environment in the family, was used in this study. FNPA was developed by Ihmels *et al*^11^ and adapted to Turkish by Ekici *et al.*^15^ The score obtained from this scale varies between 20 and 80. A higher score indicates lower risk family practices and child behaviours for childhood obesity.

The CFQ was used to assess parental feeding beliefs, attitudes and practices. CFQ was developed by Birch *et al*^16^ and adapted to Turkish by Erdim *et al.*^17^ This scale evaluates 31 items on a 5-point Likert scale, with a higher score indicating stronger perceptions, concerns, attitudes or practices. The first four of the seven subscales evaluate parental perceptions and concerns, and the last three subscales evaluate parental attitudes and practices for child feeding.^16 17^

### Statistical analyses

All statistical analyses were performed with SPSS (V.27.0), GraphPad Prism (V.10.6.1), and R (V.4.5.2). The results were presented according to data type, and parametric test assumptions mean (SD), median (IQR) and number (percentage). Numerical variables were compared across child BMI classification groups using the Kruskal-Wallis test, and pairwise comparisons were performed using Dunn’s post hoc test, taking the obesity group as the reference category. Data distribution was visualised using violin plots, which illustrate the median, IQR and the underlying probability density of the scores across groups. Multiple linear regression analysis was performed to evaluate the associations between various independent variables and child BMI-for-age percentiles. The analysis was conducted in two stages: Model 1 included primary demographic predictors (parental BMI and education levels and number of siblings), while model 2 was further adjusted for child feeding practices (CFQ subscales) and family nutrition/physical activity scores (FNPA total). Predictors were selected based on their theoretical relevance and established associations in the literature.^18^ To further explore potential non-linear associations, restricted cubic splines (RCS) with three knots were modelled using the ‘*rms*’ and ‘*ggplot2*’ packages in R software.^19^ To ensure consistency with the multivariable findings, the RCS models were adjusted for the same set of covariates included in model 2, including the number of siblings, parental education, FNPA scores and CFQ domains. Statistical significance was set at p<0.05, and 95% CIs were reported for all regression coefficients.

This manuscript was prepared in accordance with the Strengthening the Reporting of Observational Studies in Epidemiology (STROBE) recommendations.

## Results

**Figure 1:**
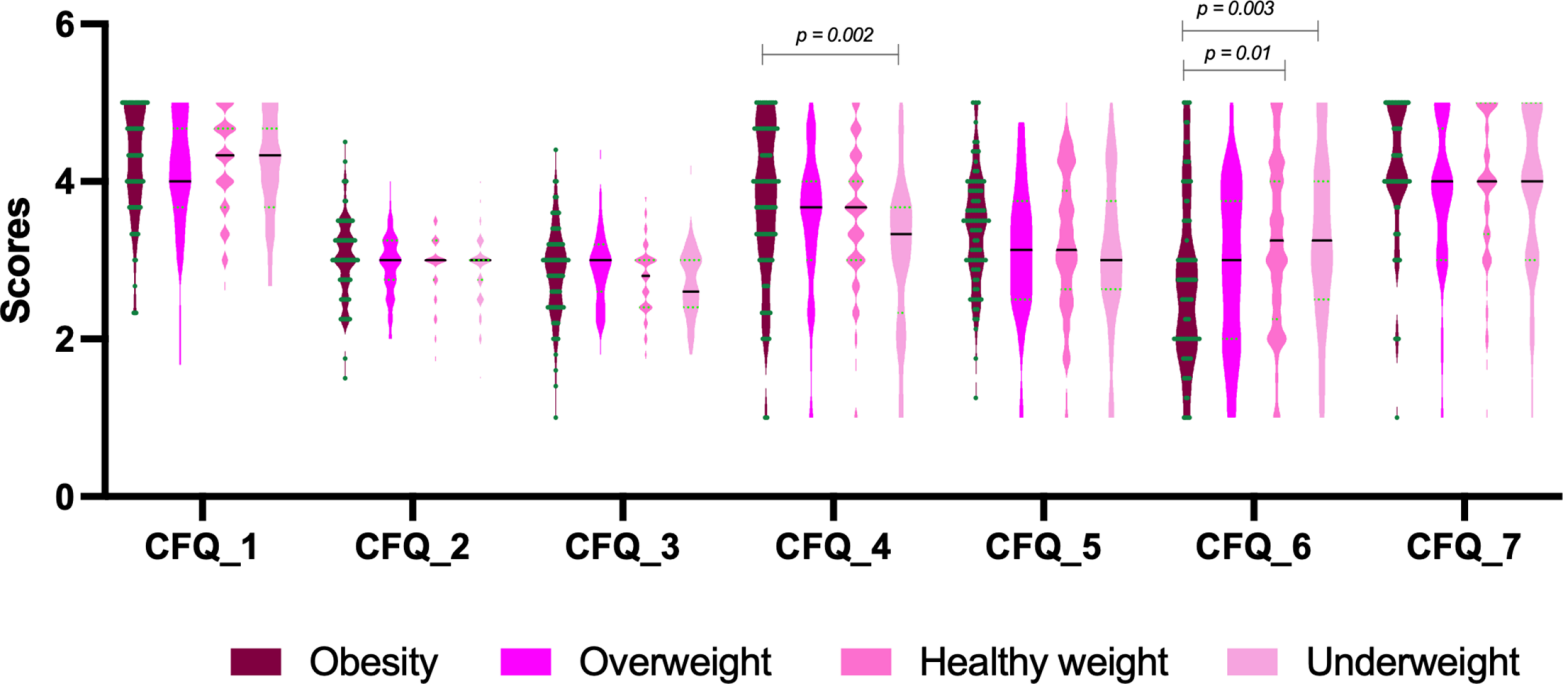
Comparison of parental feeding practices across child BMI categories. Data are visualised using truncated violin plots, where the width indicates the probability density, the solid black horizontal lines represent the medians and the dashed lines represent the IQRs. Individual data points are overlaid as green dots to illustrate the underlying distribution. Statistical significance was determined using the Kruskal-Wallis test followed by Dunn’s post hoc test for multiple comparisons. The obesity group was defined as the reference category. Statistically significant pairwise comparisons (p<0.05) are indicated with exact p values above the respective brackets. BMI, body mass index; CFQ, Child Feeding Questionnaire.

This study included 446 school-age children (ages 8 to 11) and their parents. The characteristics of the participants are presented in table 1. More than half of the mothers and children had a BMI in the healhty weight range (51.4% and 57.9%), while most of the fathers had a BMI in the overweight range (57.9%). While parental age, paternal BMI, and FNPA scores did not differ significantly across groups, maternal BMI and the number of siblings were significantly different in the obesity group compared with the other categories (p<0.05, table 2). Parental feeding practices across different child BMI categories are illustrated in figure 1. Parents in the obesity group reported significantly higher scores for ‘concern about child weight’ (CFQ-4) compared with the underweight group (p=0.002). While ‘concern’ scores in the obesity group were numerically higher than in the healthy weight and overweight groups, these differences were not statistically significant in pairwise comparisons. In contrast, ‘pressure to eat’ (CFQ-6) scores were significantly lower in the obesity group compared with both the healthy weight (p=0.01) and underweight (p=0.003) groups (figure 1). No significant pairwise differences were observed for other CFQ domains when compared with the obesity group.

**Table 1:** Characteristics of children and parents (n=446)

	Child-parent dyads
Age (years), mean±SD	9.00 (2.00)
Child female gender (n, %)	240 (53.80)
Number of siblings, mean±SD	1.34±0.82
No siblings (n, %)	45 (10.08)
1 (n, %)	243 (54.48)
2 (n, %)	122 (27.35)
>3 (n, %)	36 (8.07)
BMI classification* (n, %)	
Underweight (<5th percentile)	40 (8.97)
Healthy weight (5th to <85th percentile)	258 (57.85)
Overweight (85th to <95th percentile)	67 (15.02)
Obesity (>95th percentile)	81 (18.16)
Fat mass (%)	18.25 (11.52)
Fat free mass (kg), median (IQR)	26.55 (8.23)
Total body water (%), median (IQR)	59.70 (8.42)
Maternal age (years), mean±SD	38.55±5.62
Maternal BMI (kg/m^2^), mean±SD	25.45±4.15
Underweight (n, %)	8 (1.79)
Healthy weight (n, %)	229 (51.35)
Overweight (n, %)	147 (32.96)
Obesity (n, %)	62 (13.90)
Paternal age (years), mean±SD	42.41±5.75
Paternal BMI (kg/m^2^), mean±SD	25.59±3.12
Underweight (n, %)	2 (0.45)
Healthy weight (n, %)	133 (29.82)
Overweight (n, %)	258 (57.85)
Obesity (n, %)	53 (11.88)
1. FNPA Total Score (Nutrition and Physical Activity)	56.42±6.75
2. Total nutrition	29.63±3.23
Total physical activity	26.79±3.23
CFQ	
1.Perceived responsibility	4.18±0.66
2.Perceived parent weight	2.94±0.44
3.Perceived child weight	2.78±0.48
4.Concern	3.48±0.94
5.Restriction	3.22±0.86
6.Pressure to eat	3.09±1.07
7.Monitoring	3.93±0.95

* BMI for age percentile. Data are presented as n (%), mean±SD or median (IQR).

BMI, body mass index; CFQ, Child Feeding Questionnaire; FNPA, Family Nutrition and Physical Activity.

**Table 2:** Parental characteristics and FNPA scores by child BMI categories (n=44*6*)

	Underweight (n=40)	Healthy weight (n=258)	Overweight (n=67)	Obesity (n=81)	P value
Maternal age (years)	38.85±5.93	38.49±5.75	38.69±5.46	38.33±5.33	0.962
Paternal age (years)	43.28±6.99	42.35±5.68	42.08±5.65	42.48±5.43	0.764
Maternal BMI (kg/m^2^)	24.84±4.27*	25.15±4.14*	24.93±3.24*	27.09±4.41	**0.001**
Paternal BMI (kg/m^2^)	26.00±3.40	26.47±2.81	26.52±3.21	27.34±3.67	0.086
Siblings, mean±SD	2.40±0.71	2.43±0.86*	2.24±0.74	2.15±0.78	**0.030**
FNPA total, X̅ ±SD	56.78±7.48	56.33±6.61	56.19±6.34	56.72±7.05	0.944
1. Total nutrition	30.35±3.10	39.59±3.00	29.43±3.35	29.58±3.87	0.513
2. Total physical activity	26.43±5.29	26.75±4.69	26.76±4.66	27.14±4.63	0.874

ANOVA with two-sided Dunnett’s post hoc test (reference group: obesity) was performed.

Groups symbolised by an asterisk (*) have significant differences with the reference group (obesity) in the same line, according to pairwise comparisons. Bold values indicate statistical significance at p<0.05.

ANOVA, analysis of variance; BMI, body mass index; FNPA, Family Nutrition and Physical Activity.

Factors associated with child BMI percentiles are presented in table 3. According to the adjusted model, the most prominent association with child BMI was observed for the ‘pressure to eat’ (CFQ-6) score, which showed a significant negative association (β=−0.218, p<0.001). This was followed by the number of siblings, which remained significantly and negatively associated with child BMI (β=−0.192, p<0.001). Regarding maternal factors, higher maternal BMI was significantly associated with child BMI (β=0.136, p=0.004), whereas maternal education showed no such significant association in the adjusted model (β=−0.100, p>0.05). Interestingly, paternal education showed a significant positive association (β=0.104, p=0.040), whereas paternal BMI showed no significant association after adjustments (β=0.078, p>0.05). Among feeding practices, higher scores for ‘perceived child weight’ (CFQ-3) and ‘concern about child weight’ (CFQ-4) were also identified as factors positively associated with child BMI (β=0.143, p=0.002 and β=0.133, p=0.006, respectively). In contrast, total FNPA scores were not significantly associated with child BMI in this model (β=−0.038, p>0.05).

**Table 3:** Multiple linear regression analysis of factors associated with child BMI-for-age percentiles (n=446)

Predictors	Model 1 (R^2^: 0.048)			Model 2 (R^2^: 0.119)		
	B (95% CI)	Standardised β	P value	B (95% CI)	Standardised β	P value
Maternal BMI (kg/m^2^)	1.10 (0.34 to 1.87)	0.133	0.005	1.12 (0.36 to 1.89)	0.136	**0.004**
Maternal education (years)	−0.56 (−1.57 to 0.44)	−0.059	0.269	−0.96 (−1.94 to 0.03)	−0.100	0.057
Paternal BMI (kg/m^2^)	1.07 (0.06 to 2.08)	0.097	0.038	0.86 (−0.12, 1.84)	0.078	0.084
Paternal education (years)	0.94 (−0.14 to 2.02)	0.090	0.087	1.10 (0.05 to 2.15)	0.104	**0.040**
Number of siblings	−9.78 (−14.89 to −4.68)	−0.179	<0.001	−10.51 (−15.51 to −5.50)	−0.192	**<0.001**
CFQ-3. Perceived child weight	–	–	–	10.13 (3.87 to 16.40)	0.143	**0.002**
CFQ-4. Concern	–	–	–	4.84 (1.38 to 8.29)	0.133	**0.006**
CFQ-6. Pressure to eat	–	–	–	−6.91 (−10.03 to −3.78)	−0.218	**<0.001**
FNPA total	–	–	–	−0.19 (−0.70 to 0.31)	−0.038	0.451

Multiple Regression analysis was performed

R2, adjusted R square. Model 1 is the crude model. Model 2. Adjusted for scores of CFQ sub-scales and total score of FNPA. Bold values indicate statistical significance at p<0.05.

BMI, body mass index; CFQ, Child Feeding Questionnaire; FNPA, Family Nutrition and Physical Activity.

**Figure 2:**
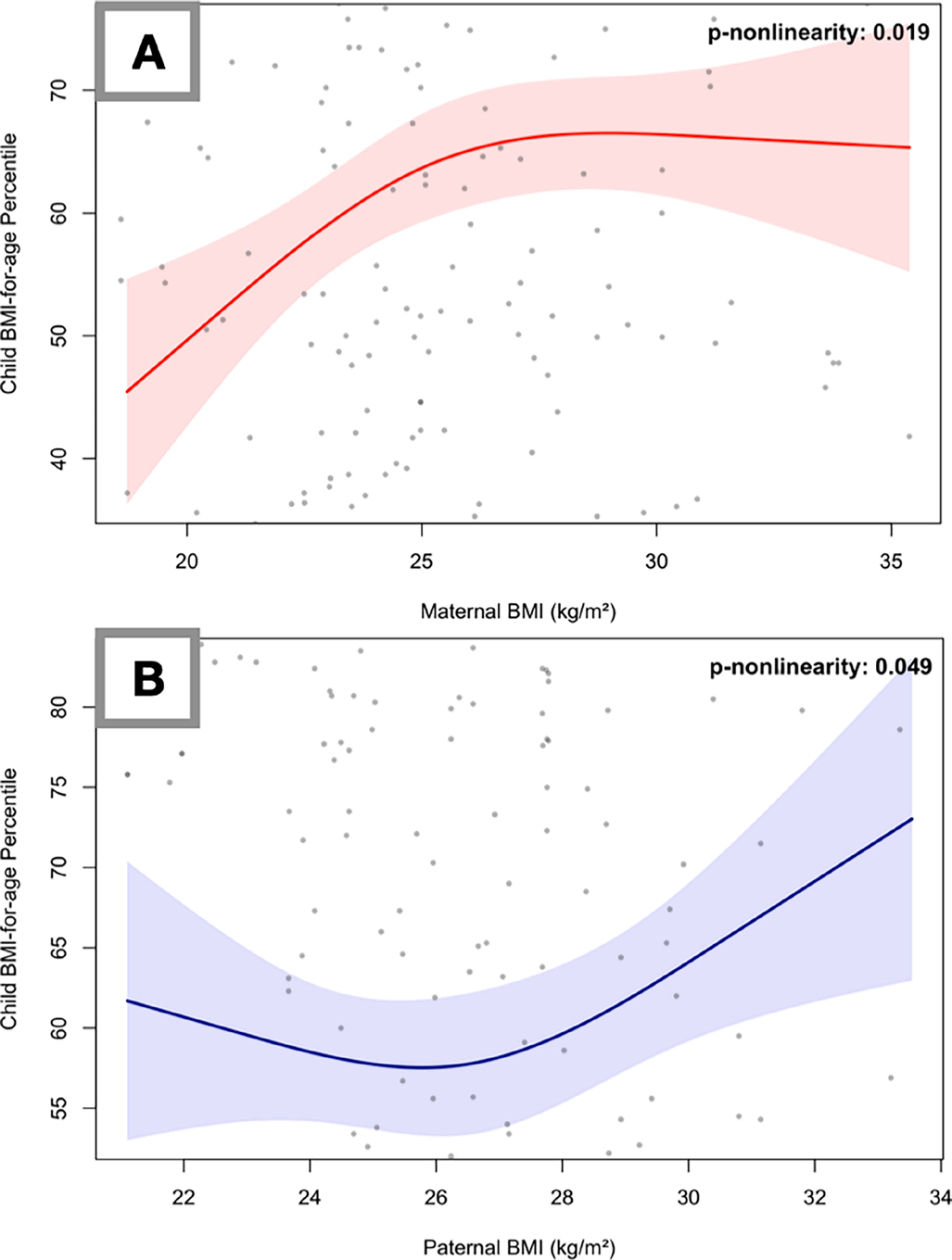
Restricted cubic spline plots illustrating the adjusted associations between parental BMI and child BMI-for-age percentiles. (A) Maternal BMI and (B) paternal BMI. The solid lines (red for maternal, dark blue for paternal) represent the predicted mean percentiles, and the shaded areas indicate the 95% CIs. Models are adjusted for all covariates included in model 2 (number of siblings, maternal and paternal education, family nutrition and physical activity scores and all child feeding domains). Grey dots represent the observed individual data points. BMI, body mass index.

Potential associations between parental and child BMI were further explored using RCS analysis (figure 2). A significant non-linear pattern was observed for maternal BMI (pnonlinearity**=**0.019), where an initial increase in child BMI percentiles tended to level off at higher BMI values (figure 2A). Similarly, the association for paternal BMI was found to be non-linear (pnonlinearity**=**0.049), with the positive relationship becoming more pronounced at higher paternal BMI levels (figure 2B).

## Discussion

This study evaluated the complex associations between family environment dynamics, parental feeding practices, and childhood obesity in parent–child dyads. The results revealed a distinct dichotomy: while general familial factors, including parental age, paternal BMI, and total FNPA scores, showed no significant variance across BMI groups, specific maternal and feeding behaviour variables demonstrated strong associations with childhood obesity. In the current study, higher maternal BMI, along with elevated scores for ‘perceived child weight’ and ‘parental concern’, was significantly associated with higher child BMI percentiles, whereas ‘pressure to eat’ showed a significant inverse relationship. Notably, parental BMI followed divergent patterns, where the association levelled off at higher maternal BMI levels but became more pronounced for fathers as BMI increased. Ultimately, this study contributes original insights by simultaneously integrating sociodemographic characteristics with feeding-related attitudes. In contrast to earlier studies that narrowly address isolated nutritional practices,^20 21^ the current research offers a robust, multifaceted model that highlights the critical intersection of both sociodemographic and behavioural influences. A visual summary of the study design and principal findings is provided in (online supplemental graphical abstract).

The higher maternal BMI observed in children with obesity in this study (table 2) has been well described previously in the literature.^22^ Higher maternal BMI may be associated with childhood obesity through both genetic and environmental pathways. Mothers play a key role in shaping their children’s eating habits and weight outcomes by influencing the home food environment and modelling dietary preferences, which may be associated with a higher likelihood of obesity in children.^22^ Also, maternal BMI has been linked to children’s eating behaviours and physical activity routines,^23^ and Linabery *et al*^24^ found that maternal obesity had a stronger link to infant BMI compared with paternal obesity. Our findings indicate that each 1 kg/m² increase in maternal BMI is associated with a 1.12-unit increase in the child’s BMI percentile, suggesting a positive overall relationship. However, further analyses revealed a divergent and non-linear pattern in how parental BMI relates to child BMI-for-age percentiles, particularly when approaching the obesity threshold (30 kg/m²). While maternal BMI appeared to reach a plateau at higher BMI levels, the paternal association exhibited a more pronounced upward trend beyond this point. This divergent pattern may reflect fundamental differences in how maternal versus paternal BMI translates into child weight outcomes. The maternal plateau may suggest a ceiling effect, whereby the behavioural and environmental influences already described reach a point of saturation beyond which additional increases in maternal BMI confer diminishing marginal risk.^22 25^ In contrast, the U-shaped paternal association may point toward a threshold effect driven more by shared genetic predisposition or household lifestyle factors than by direct caregiving behaviours.^24^ These non-linear patterns warrant cautious interpretation given the wide CIs observed, particularly at the extremes of the paternal BMI distribution.

In the current study, the importance of maternal characteristics and feeding practices in relation to childhood obesity/overweight was further highlighted (figure 1 and table 3), supporting the view that maternal approaches to feeding may be associated with early childhood weight gain and obesity-promoting eating behaviours.^26^ The finding that higher maternal BMI was associated with childhood obesity/overweight is consistent with the literature.^22 27^ On the other hand, previous research on the effects of mothers’ education has reported varying results. While Muthuri *et al*^27^ reported that higher maternal education levels were associated with childhood overweight, other findings have shown that lower maternal education is linked to an increased likelihood of higher BMI in children.^28^

Interestingly, paternal BMI was not significantly associated with childhood obesity after adjustment. This finding contrasts with evidence from systematic reviews and meta-analyses reporting that higher paternal BMI is generally associated with increased obesity risk in children.^29 30^ Several explanations may account for this discrepancy. First, mothers typically assume greater responsibility for childcare and feeding routines, serve as primary role models for eating behaviours, and spend more time with children daily; moreover, cultural norms that assign primary caregiving responsibilities to mothers may overshadow paternal influences^30 31^—all of which may amplify the influence of maternal BMI relative to paternal BMI on child weight outcomes. Second, collinearity between maternal and paternal BMI in the adjusted model may have attenuated the independent association of paternal BMI, making it harder to detect a statistically significant effect. Together, these factors may explain why paternal BMI lost significance on adjustment, despite its established role in the broader literature.

As close members of a child’s social environment, siblings play a crucial role in shaping their social, emotional, and physical development.^32^ Children’s weight status may be affected by the number of siblings, as this can influence both the amount of parental time and attention each child receives and the dynamics of sibling interactions.^33^ In the present cross-sectional study, a higher number of siblings was associated with lower child BMI percentiles. This finding aligns with previous research, which consistently indicates that a higher number of siblings corresponds to significantly lower odds of excessive weight gain in children.^34^ Having more siblings may be associated with a lower likelihood of childhood obesity. In larger families, parental attention and resources are divided, which may limit overfeeding and indulgent behaviours. Siblings often model healthy behaviours and engage in shared physical activities, promoting active lifestyles. Additionally, food may be more evenly distributed, leading to smaller portions and more structured meals. Children with siblings also tend to be more physically active and spend less time in sedentary activities.^35^

Previous research has shown a consistent inverse association between FNPA scores and both adiposity and BMI in children and adolescents with overweight or obesity.^36 37^ Despite prior studies demonstrating a significant inverse association between FNPA scores and BMI in children, our findings did not replicate this relationship. This discrepancy may be due to factors such as genetics, pubertal stage, psychosocial stress, or medical conditions that influence BMI independently of the family environment or behaviours assessed by the FNPA.

Four subdimensions (Perceived Responsibility, Perceived Parent Weight, Perceived Child Weight, and Concern about Child Weight) of CFQ evaluate parents’ views and worries regarding their child’s potential risk of obesity. The remaining three subdimensions—Restriction, Pressure to Eat and Monitoring—reflect parental strategies related to controlling feeding behaviours.^38^ In the context of feeding practices, higher scores on the ‘perceived child weight’ (CFQ-3) and ‘concern about child weight’ (CFQ-4) subscales were associated with higher child BMI. These findings may suggest that parents’ perceptions and concerns tend to increase because of the child’s elevated weight status, rather than acting as independent causal antecedents—a classic pattern of reverse causality in cross-sectional designs. These significant results are in line with previous studies, which have consistently shown that mothers of children with obesity tend to express greater concern about their child’s weight.^15^ Many previous studies have reported that parents’ perceptions (especially regarding perceived child weight) show significant sociocultural differences and may be an important factor in childhood obesity.^39 40^

In the present study, ‘pressure to eat’ (CFQ-6) scores were lower among children with obesity compared with both healthy weight and underweight groups. Consistently, in the adjusted model, ‘pressure to eat’ emerged as the most prominent factor associated with child BMI, showing a significant inverse relationship. Taken together, these findings suggest that parents may be less likely to encourage food intake in children with higher BMI, which may reflect a response to the child’s current weight status rather than a causal influence. Importantly, the cross-sectional nature of this study precludes causal inference; it is plausible that the child’s weight status shapes parental feeding behaviours and perceptions—including perceived child weight (CFQ-3), concern (CFQ-4), and pressure to eat (CFQ-6)—rather than the reverse.

### Limitations and strengths

This study has several limitations that should be acknowledged. First, the reliance on a volunteer-based sample may introduce potential selection bias, which could limit the generalisability of our findings. Second, although our models adjusted for key familial and behavioural variables, the potential for residual confounding remains, as data on other important unmeasured factors—such as the explicit quantification of children’s dietary intake, physical activity levels, screen time and pubertal status—were not available. Third, the reliance on self-reported questionnaires inherently introduces the risk of recall bias. Furthermore, although complete data were obtained for the child-mother-father triads, parents did not participate equally in the face-to-face interviews. Mothers were generally more active in responding, meaning the reported feeding behaviours and family dynamics may predominantly reflect the maternal perspective. As a fourth limitation, although body composition variables (fat mass, fat-free mass and total body water) were measured to provide a more detailed assessment of nutritional status, they were not included in the main analyses. Finally, the cross-sectional nature of the study precludes any causal inferences, and the single-city sample may further limit the generalisability of the findings to other populations. Despite these limitations, a major strength of this research lies in its robust, multifaceted examination of how interconnected parental sociodemographic factors, feeding behaviours and obesogenic environments collectively influence childhood obesity. Additional strengths include its important public health focus, adequate sample size and the comprehensive use of multiple validated questionnaires.

These findings carry practical implications for family-based obesity prevention and counselling. Clinicians and dietitians working with families of children with obesity should be aware that parental concern and perception of child weight may already be elevated as a reactive response, rather than a modifiable risk factor. Accordingly, counselling strategies may benefit from redirecting focus toward concrete behavioural targets—such as reducing pressure to eat and establishing structured, responsive feeding practices—rather than solely addressing parental awareness of child weight status.

## Conclusions

This study contributes original evidence by simultaneously integrating sociodemographic characteristics of parents with parental feeding-related attitudes and behaviours to better understand their collective associations with childhood obesity in parent-child dyads. These findings highlight potentially modifiable correlates of childhood obesity—particularly maternal BMI, parental feeding perceptions and pressure to eat—and may inform the development of family-centred health promotion strategies that consider both parental weight status and adaptive feeding practices. Further observational and interventional studies are needed to establish direction and identify effective parental approaches and relevant risk characteristics within diverse sociocultural contexts.

## Supplementary material

10.1136/bmjpo-2026-004819 online supplemental file 1

## Data Availability

The datasets generated during and/or analysed during the current 13 study are available from the corresponding author on reasonable request.
