# Supplementary figures and images for: Role of parental characteristics and feeding practices in childhood obesity: a cross-sectional study
Source: BMJ Paediatr Open. 2026 Jun 4;10(1):e004819. doi: 10.1136/bmjpo-2026-004819 (PMC13239359; doi:10.1136/bmjpo-2026-004819)

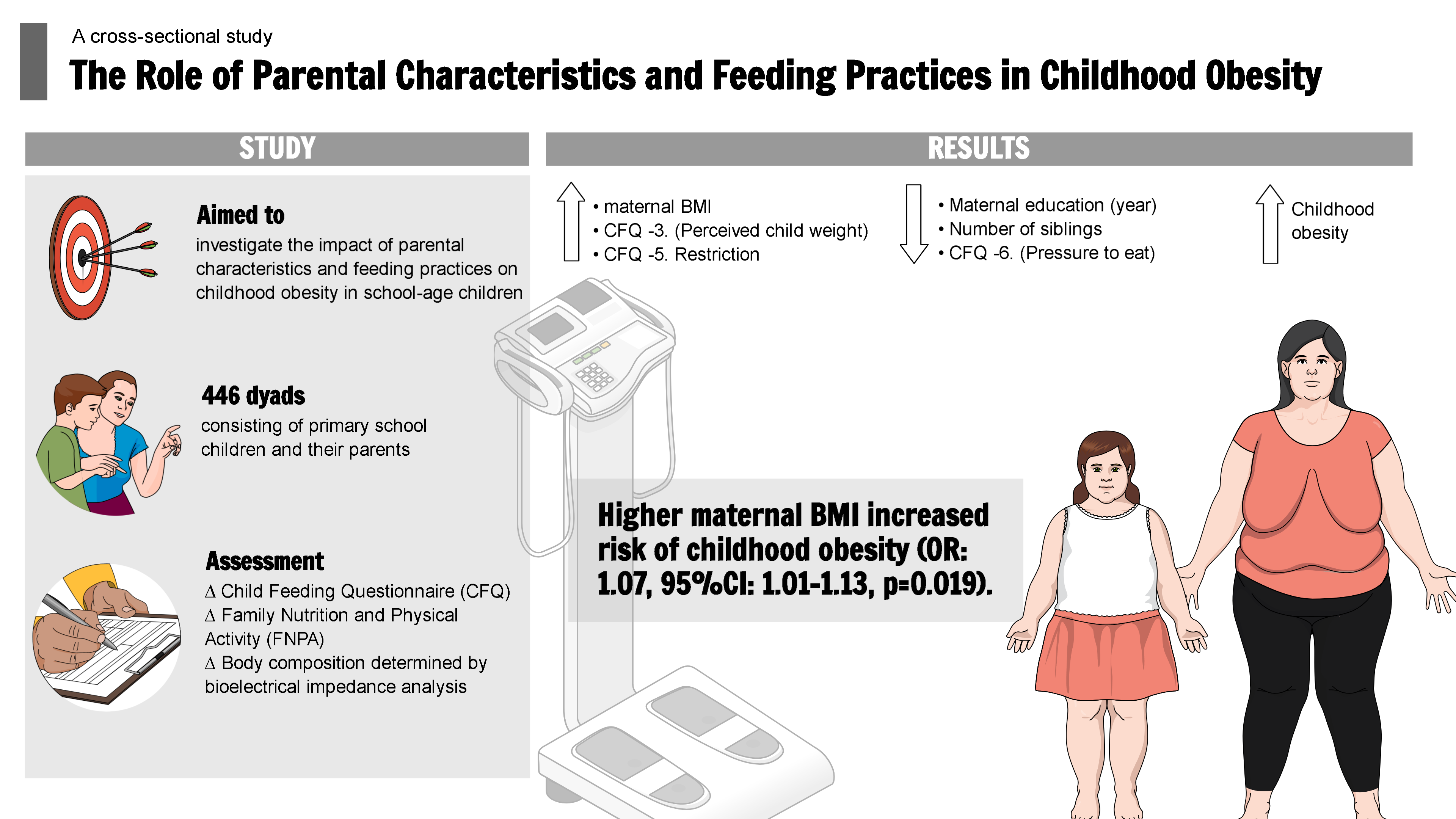

Supplement: online supplemental file 1 [file bmjpo-10-1-s001.png]
