# Supplementary material for: Role of parental characteristics and feeding practices in childhood obesity: a cross-sectional study
Source: BMJ Paediatr Open. 2026 Jun 4;10(1):e004819. doi: 10.1136/bmjpo-2026-004819 (PMC13239359; doi:10.1136/bmjpo-2026-004819)
Supplement: Supplementary file 2 [file bmjpo-2026-004819.reviewer_comments.pdf]

PEER REVIEW HISTORY

BMJ Paediatrics Open publishes all reviews undertaken for accepted manuscripts. Reviewers are asked to complete a checklist review form and are provided with free text boxes to elaborate on their assessment. These free text comments are reproduced below.

ARTICLE DETAILS

**TITLE (PROVISIONAL)**	The Role of Parental Characteristics and Feeding Practices in Childhood Obesity: A Cross-Sectional Study
**AUTHORS**	Önal, Seda<br>ŞİMŞEK, Hilal<br>Uçar, Aslı

VERSION 1 – REVIEW

**REVIEWER NAME**	Flom, Peter
**REVIEWER AFFILIATION**	Peter Flom Consulting
**REVIEWER CONFLICT OF INTEREST**	No Conflict of Interest
**AI DISCLOSURE**	No, I have not used AI
**DATE REVIEW RETURNED**	17-Apr-2026

**GENERAL COMMENTS**	I confine my remarks to statistical and methodological aspects of this paper. Unfortunately, there are some major issues that need to be addressed before I can recommend publication.<br><br>The biggest issue is the dichotimization of the dependent variable. BMI is problematic in itself, although it is widely used. See e,g, my article on Medium https://medium.com/peter-flom-the-blog/why-bmi-is-a-bad-measure-of-obesity-and-what-is-better-f8a62fc9ca49 or many other articles. Categorizing makes it worse. Categorizing a continuous variable increases type I and type !! error and introduces a kind of "magical thinking" - that something special happens at the cutpoint.<br><br>Please discuss the flaws of BMI and say why you did not use a better measure.<br><br>Then, rather than using logistic regression, leave BMI continuous and use regular regression with a spline of BMI, since the effects are likely to be nonlinear and maybe even nonmonotonic.<br><br>Other issues: The figure is a dynamite plot. These are not recommended. See e.g. this open letter https://simplystatistics.org/posts/2019-02-21-dynamite-plots-must-die/ or many other articles.<br><br>If you insist on categorizing BMI, at least use four categories and ordinal logistic regression.<br><br>The sample consists of volunteers. This adds potential bias to the results. This needs to be discussed in limitations.<br><br>Sorry to be so negative.

**REVIEWER NAME**	Bansal, Utkarsh
**REVIEWER AFFILIATION**	
**REVIEWER CONFLICT OF INTEREST**	No Conflict of Interest
**AI DISCLOSURE**	No, I have not used AI
**DATE REVIEW RETURNED**	22-Apr-2026

**GENERAL COMMENTS**	Thank you for the opportunity to review this manuscript.<br>The study addresses an important public health topic and uses an adequate sample size with validated questionnaires. The manuscript is relevant and has potential value; however, few issues require clarification and revision.<br><br>1. Title and Abstract (Page 1)<br>The title is clear and appropriately reflects the cross-sectional design, and the abstract is structured and informative.<br>Page 1, Line 44: The combined prevalence of overweight and obesity (33.2%) is reported without separately stating the prevalence of obesity alone. Please provide the isolated obesity prevalence for clarity.<br>Page 1, Lines 47–48: As this is a cross-sectional study, causal inferences should be avoided. Statements such as “higher maternal BMI increased the risk of obesity” should be revised to wording such as “was associated with higher odds.”<br>Similar corrections should be made throughout the Abstract, Results, Discussion, and Conclusion where relevant.<br><br>2. Introduction (Pages 2-3)<br>The introduction provides a good global background with recent data of childhood obesity. However, the specific research gap is not clearly defined.<br>The rationale for combining CFQ, FNPA, and parental characteristics should be explained more clearly, particularly what new knowledge this study adds beyond previous CFQ-based or parental feeding studies.<br>3. Methods<br>The sample size appears adequate (Page 3, Line 41; n=446).<br>Page 3, Line 39: Grammatical error: “attending in primary school” should be revised to “attending primary school.”<br>(Page 4, Line 3). Some methodological details need clarification, including recruitment method, sampling technique (random vs convenience) school selection process, whether one or both parents were interviewed, and handling of missing data<br>Important confounders such as dietary intake, physical activity, screen time, and pubertal status do not appear to have been considered and should be justified or acknowledged as limitations.<br>(Page 4, line 16-18). The manuscript states WHO Anthro software version 3.2 was used. WHO Anthro is usually for children under 5 years; school-age 8–11 often requires WHO AnthroPlus. Please verify software used.<br>Page 4, Lines 12–13: Body composition variables (fat mass, fat-free mass, total body water) were measured but not meaningfully analysed. Please justify their inclusion or mention this as a limitation.

	4. Statistical Analysis<br>Page 4, Lines 54–55: Further clarification of the regression model is needed, including which variables were entered, whether any selection criteria were used,<br>Page 4, Line 47: The sentence “results were represented according to the data type” should be revised to “results were presented according to data type.”<br>5. Results<br>Please report the isolated prevalence of obesity in addition to the combined prevalence reported in the abstract.<br>Maternal BMI was statistically significant (OR 1.07), but the effect size is modest and would benefit from clearer clinical interpretation.<br>Please also state the number of complete cases included in Table 3 and clarify whether missing data were present in regression analyses.<br>Why overweight and obesity were combined into one outcome. Combining overweight and obesity into a single category may mask important differences Please justify this decision or provide subgroup analysis if feasible<br>Table-specific comments:<br>• Table 1, Line 21: “Health weight” should read “Healthy weight.”<br>• Table 1, Line 27: Fat-free mass median (IQR) of 26.55 (52.20) appears unusual and should be rechecked. How can be an IQR be more than median.<br>• Tables 1: Please align decimal places consistently.<br>• Table 2: “0086” likely should be “0.086.”<br><br>6. Ethics<br>There is an inconsistency regarding ethics approval:<br>Page 5, Lines 10–11: Fırat University Ethics Commission<br>Page 9, Lines 38–39: Ankara University Ethics Commission<br>This discrepancy should be corrected.<br>7. Discussion<br>The Discussion summarises the findings appropriately, but some interpretations are stronger than justified for a cross-sectional study. Findings should be described as associations only.Page 6, Lines 24–26: The phrase “higher maternal BMI was associated higher risk” should be revised grammatically and conceptually to “was associated with higher odds.”<br>Page7, line 17-18 : The role of paternal BMI was not significant after adjustment. This finding deserves discussion, including potential explanations such as maternal influence on feeding patterns, caregiving roles, or collinearity<br>Page8, line 41-42 : The possibility of reverse causality should be discussed, particularly regarding restrictive feeding practices.<br>Clinical implications may be expanded by explaining how these findings could inform family-based obesity prevention or counselling strategies.<br><br>8. References, Limitations and Conclusion

	Page 9, Lines 3–7: The limitations are mentioned but should more clearly include the recall bias from self-reported questionnaires, lack of dietary quantification, residual confounding, and limited generalisability from a single-city sample.<br>Page 9, Lines 7–9: Additional strengths may be highlighted, including the important public health focus, adequate sample size, and comprehensive use of multiple validated questionnaires.<br>Page 9, Line 12: The conclusion should remain balanced and aligned with the study design, avoiding statements that imply prediction or causation.<br>Some WHO references appear incorrect or non-specific (e.g., Reference 14)<br>Full reference list should be reviewed once.

VERSION 1 – AUTHOR RESPONSE

REVIEWER 1 COMMENTS

I confine my remarks to statistical and methodological aspects of this paper. Unfortunately, there are some major issues that need to be addressed before I can recommend publication.

We thank the reviewer for their evaluation of the statistical and methodological framework of our study. In response to the concerns raised, we have both refined our existing analyses and incorporated additional statistical methods to ensure the robustness of our results. Our point-by-point responses are presented below.

1. The biggest issue is the dichotimization of the dependent variable. BMI is problematic in itself, although it is widely used. See e,g, my article on Medium https://medium.com/peter-flom-the-blog/why-bmi-is-a-bad-measure-of-obesity-and-what-is-better-f8a62fc9ca49 or many other articles. Categorizing makes it worse. Categorizing a continuous variable increases type I and type II error and introduces a kind of "magical thinking" - that something special happens at the cutpoint. Please discuss the flaws of BMI and say why you did not use a better measure.
2. Then, rather than using logistic regression, leave BMI continuous and use regular regression with a spline of BMI, since the effects are likely to be nonlinear and maybe even nonmonotonic.

- *We thank the reviewer for this critical suggestion. In accordance with this recommendation, we have replaced the previous logistic regression model with Multiple Linear Regression (MLR), using child BMI-for-age percentiles as a continuous outcome. Table 3 has been entirely updated (B coefficients, 95% CIs, and standardized β). All associated content throughout the main manuscript has been revised accordingly to reflect these methodological changes.*
- *Additionally, to address the potential for non-linear or non-monotonic associations, we performed Restricted Cubic Spline (RCS) analysis for parental BMI variables. The results of these advanced analyses are now presented in Figure 2. We firmly believe that this valuable recommendation has substantially improved the overall quality and depth of our manuscript, and we sincerely thank the reviewer.*

3. The figure is a dynamite plot. These are not recommended. See e.g. this open letter https://simplystatistics.org/posts/2019-02-21-dynamite-plots-must-die/ or many other articles.

- *We sincerely thank the reviewer for this valuable methodological feedback. Following your recommendation, we have completely redesigned Figure 1. It is now presented as a truncated violin plot. This updated visualization provides full data transparency by displaying the probability density, the median scores (solid black lines), and the overlaid individual data points (green dots) for all participants. We believe this significantly enhances the methodological rigor of our data presentation. The corresponding figure legend has also been updated to reflect these changes.*

4. If you insist on categorizing BMI, at least use four categories and ordinal logistic regression.

- *In line with the reviewer's previous suggestion, we have completely removed BMI categorization in favor of continuous BMI within the multiple linear regression (Table 3).*

5. The sample consists of volunteers. This adds potential bias to the results. This needs to be discussed in limitations.

- *Thank you for highlighting this important limitation. To address this, the limitations paragraph has been revised to include a discussion on the potential bias introduced by our volunteer sample. (page 10, lines 2-4)*

REVIEWER 2 COMMENTS

Thank you for the opportunity to review this manuscript. The study addresses an important public health topic and uses an adequate sample size with validated questionnaires. The manuscript is relevant and has potential value; however, few issues require clarification and revision.

1. Title and Abstract (Page 1): The title is clear and appropriately reflects the cross-sectional design, and the abstract is structured and informative.

- *We thank the reviewer for their valuable comments. Our point-by-point responses to each specific comment are provided below.*

2. Page 1, Line 44: The combined prevalence of overweight and obesity (33.2%) is reported without separately stating the prevalence of obesity alone. Please provide the isolated obesity prevalence for clarity.

- *We thank the reviewer for this suggestion. Accordingly, the prevalence of obesity has been added to the abstract. (Page 1, line 23)*

3. Page 1, Lines 47–48: As this is a cross-sectional study, causal inferences should be avoided. Statements such as “higher maternal BMI increased the risk of obesity” should be revised to wording such as “was associated with higher odds.”
   Similar corrections should be made throughout the Abstract, Results, Discussion, and Conclusion where relevant.

- *We thank the reviewer for this critical suggestion. Accordingly, we have revised the language throughout the main manuscript to avoid causal inferences, ensuring that the associations are described appropriately for the study design. Furthermore, in line with the statistical recommendations from Reviewer 1, the Results section has been updated to reflect the revised analyses (In abstract, (Page1, lines 23-31).*

Introduction (Pages 2-3)

4. The introduction provides a good global background with recent data of childhood obesity. However, the specific research gap is not clearly defined.

- *We thank the reviewer for this valuable comment. In response, we have revised the Introduction to more clearly articulate the specific research gap. While previous studies have predominantly examined parental feeding practices, parental BMI, and family environment separately, their relative contributions and potential non-linear associations with child BMI remain insufficiently understood. Additionally, the interplay between distinct family environment factors and parental feeding behaviours has been underexplored in the current literature. To address this gap, we have explicitly emphasised the need for an integrated analytical framework that simultaneously considers socio-demographic, behavioural, and anthropometric factors. Accordingly, the final paragraph of the Introduction has been revised to highlight the novelty and objective of our study more clearly. (page 3, lines 7-17)*

5. The rationale for combining CFQ, FNPA, and parental characteristics should be explained more clearly, particularly what new knowledge this study adds beyond previous CFQ-based or parental feeding studies.

- *We thank the reviewer for this valuable comment. The Introduction has been revised to clarify the rationale for combining CFQ, FNPA, and parental characteristics. Previous studies have largely examined these factors separately, despite their interrelated nature and potential joint influence on child BMI. By integrating feeding practices, family environment, and parental anthropometric characteristics within a single framework, our study provides a more comprehensive evaluation of their combined and relative contributions. In addition, the use of continuous BMI outcomes and advanced modelling allows the identification of potential non-linear associations not addressed in earlier CFQ-based or single-domain studies. (page 3, lines 9-13)*

Methods:

6. The sample size appears adequate (Page 3, Line 41; n=446).
- *We thank the reviewer for this comment. However, we would like to highlight a significant revision: due to the refinement of our regression models, the sample size calculation—originally based on logistic regression—has been updated to align with the multiple linear regression (MLR) analysis. This ensures that the reported power calculations are consistent with the primary statistical methods used in the study. (Page 3-4, lines 30 and 1-3, respectively).*
7. Page 3, Line 39: Grammatical error: "attending in primary school" should be revised to "attending primary school."
- *We thank the reviewer for this comment. This typo has been revised accordingly. (Page 3, line 20)*
8. (Page 4, Line 3). Some methodological details need clarification, including recruitment method, sampling technique (random vs convenience) school selection process, whether one or both parents were interviewed, and handling of missing data.
- *We thank the reviewer for pointing out these important issues. In accordance with this suggestion, the first paragraph of the 'Materials and Methods' section has been thoroughly revised and rewritten to provide greater clarity. (Page 3, lines 21-27).*
9. Important confounders such as dietary intake, physical activity, screen time, and pubertal status do not appear to have been considered and should be justified or acknowledged as limitations.
- *Thank you for highlighting this important limitation. To address this, the limitations paragraph has been revised. (Page 10, lines 4-7).*
10. (Page 4, line 16-18). The manuscript states WHO Anthro software version 3.2 was used. WHO Anthro is usually for children under 5 years; school-age 8–11 often requires WHO AnthroPlus. Please verify software used.
- *We appreciate the reviewer's careful reading and the opportunity to rectify this oversight. The software name and version were indeed incorrectly reported and have been revised accordingly in the updated text. (Page 4, line 13).*
11. Page 4, Lines 12–13: Body composition variables (fat mass, fat-free mass, total body water) were measured but not meaningfully analysed. Please justify their inclusion or mention this as a limitation.
- *We thank the reviewer for this valuable comment. Although body composition variables (fat mass, fat-free mass, and total body water) were measured to provide a more detailed assessment of nutritional status, they were not included in the main analyses. This has now been acknowledged as a limitation of the study in the revised manuscript. (Page 10, lines 11-14).*
12. Statistical Analysis: Page 4, Lines 54–55: Further clarification of the regression model is needed, including which variables were entered, whether any selection criteria were used,
- *The section regarding the regression model has been further detailed and revised in alignment with the updated Table 3 (Multiple Linear Regression). We thank the reviewer for this suggestion, which has helped clarify the statistical approach used in the analysis. (please see Statistical Analyses paragraph).(Pages 4-5, lines 27-29, and 1-16, respectively)*

13. Page 4, Line 47: The sentence “results were represented according to the data type” should be revised to “results were presented according to data type.”

- *We appreciate the reviewer’s careful reading. This error has been corrected in the revised manuscript. (Page 4, line 28).*

Results:

14. Please report the isolated prevalence of obesity in addition to the combined prevalence reported in the abstract.

- *We thank the reviewer for this suggestion. Accordingly, the prevalence of obesity has been added to the abstract. (Page 1, line 23)*

15. Maternal BMI was statistically significant (OR 1.07), but the effect size is modest and would benefit from clearer clinical interpretation.

- *We agree that a clearer clinical interpretation is necessary. As previously noted, we have transitioned from LR to MLR in this revision. In the revised Model 2 (Table 3), maternal BMI showed a significant association (B=1.12, β=0.136, p=0.004), indicating that each 1 kg/m^2 increase in maternal BMI is associated with a 1.12-unit increase in the child’s BMI percentile. This modest effect size supports our primary conclusion that childhood BMI is not solely determined by maternal weight status, leaving significant room for other modifiable factors, such as parental feeding practices, which showed even stronger associations in our model. We have added this perspective to the Discussion section (page 7, lines 18-31).*

16. Please also state the number of complete cases included in Table 3 and clarify whether missing data were present in regression analyses.

- *We thank the reviewer for this comment. As stated in the 'Materials and Methods' section, only child–parent triads with complete datasets were included in the study. Consequently, the multiple linear regression analysis presented in Table 3 was conducted using 446 complete cases, with no missing data present in the final models. We have updated the title of Table 3 to reflect the sample size (n=446) for further clarity.*

17. Why overweight and obesity were combined into one outcome. Combining overweight and obesity into a single category may mask important differences Please justify this decision or provide subgroup analysis if feasible.

- *We thank the reviewer for their observation and valuable suggestion regarding this critical statistical point. In alignment with the comments from Reviewer 1, we have moved away from the categorical use of BMI (**e.g., presence of obesity/overweight vs. absence**) and have updated Table 3 accordingly. We believe these revisions, while partially altering the results, have substantially improved the overall quality and robustness of the study. Furthermore, additional analyses were performed for parental BMI variables, and the results have been incorporated into the manuscript as Figure 2.*

18. Table 1, Line 21: “Health weight” should read “Healthy weight.”

- *Thank you for noting this. This typo has been revised accordingly.*

19. Table 1, Line 27: Fat-free mass median (IQR) of 26.55 (52.20) appears unusual and should be rechecked. How can be an IQR be more than median.

- *We thank the reviewer for this careful observation. We regret that the Range (52.20) was inadvertently reported instead of the IQR. This error has been corrected in Table 1, and all other values in the table have been double-checked to ensure accuracy. We appreciate your attention to this detail.*

20. Tables 1: Please align decimal places consistently And Table 2: “0086” likely should be “0.086.”

- *Thank you for this observation. All tables have been reviewed and revised to maintain consistency in the number of decimal places for all values.*

21. There is an inconsistency regarding ethics approval: Page 5, Lines 10–11: Fırat University Ethics Commission. Page 9, Lines 38–39: Ankara University Ethics Commission. This discrepancy should be corrected.

- *Dear Editor, we are sincerely grateful to you for identifying this critical error. We believe this discrepancy resulted from a drafting error in the manuscript; accordingly, it has been revised while ensuring the Ethical Approval section in the Methods remains intact. We thank you for your careful oversight. (Page 5, lines 18-21).*

Discussion:

22. The Discussion summarises the findings appropriately, but some interpretations are stronger than justified for a cross-sectional study. Findings should be described as associations only.Page 6, Lines 24–26: The phrase “higher maternal BMI was associated higher risk” should be revised grammatically and conceptually to “was associated with higher odds.”

- *We thank the reviewer for this important comment. We agree that the interpretations should be aligned with the cross-sectional design of the study. Accordingly, the Discussion has been revised to ensure that all findings are described in terms of associations rather than causal relationships. Statements implying causality or “risk” have been carefully rephrased throughout the manuscript.*

23. Page7, line 17-18 : The role of paternal BMI was not significant after adjustment. This finding deserves discussion, including potential explanations such as maternal influence on feeding patterns, caregiving roles, or collinearity

- *We appreciate the reviewer's comment. We agree that the loss of significance of the paternal BMI after adjusting for covariates needs clarification. (This association also holds in our new fully adjusted regression model.) (page 8, lines: 10-21).*

24. Page8, line 41-42 : The possibility of reverse causality should be discussed, particularly regarding restrictive feeding practices.

- *We thank the reviewer for this important methodological point. In the original analysis, both CFQ-3 (Perceived Child Weight) and CFQ-5 (Restriction) were significantly associated with child BMI, making reverse causality particularly relevant for restrictive feeding practices. Following the reviewer's (R1) feedback, we revised our analytical model, and notably, CFQ-5 no longer reached statistical significance in the updated analysis. Nevertheless, we have expanded the discussion of reverse causality to address all significant CFQ subdimensions — specifically CFQ-3, CFQ-4, and CFQ-6 — acknowledging that the child's current weight status may itself shape both parental perceptions and feeding behaviours, rather than these practices acting as independent causal predictors. (page 9, lines:15-18 and 26-31).*

25. Clinical implications may be expanded by explaining how these findings could inform family-based obesity prevention or counselling strategies.

- *We thank the reviewer for this constructive suggestion. We have expanded the Discussion section accordingly. (page 10, lines: 21-27).*

26. Limitations and Conclusion: Page 9, Lines 3–7: The limitations are mentioned but should more clearly include the recall bias from self-reported questionnaires, lack of dietary quantification, residual confounding, and limited generalisability from a single-city sample.

- *We thank the reviewer for this helpful comment. We agree that the limitations should be more clearly specified. Accordingly, the Limitations and Conclusion section has been revised to explicitly include recall bias due to self-reported questionnaires, the lack of dietary quantification, potential residual confounding, and the limited generalisability of the findings due to the single-city sample.(Page 10, lines:5-8, and 15)*

27. Page 9, Lines 7–9: Additional strengths may be highlighted, including the important public health focus, adequate sample size, and comprehensive use of multiple validated questionnaires.

- *We thank the reviewer for this valuable suggestion. We agree that the strengths of the study can be further emphasized. Accordingly, the Limitations and Conclusion section has been revised to highlight additional strengths, including the important public health focus, adequate*

sample size, and the comprehensive use of multiple validated questionnaires. (page 10, lines: 16-20).

28. Page 9, Line 12: The conclusion should remain balanced and aligned with the study design, avoiding statements that imply prediction or causation.

- *We thank the reviewer for this valuable feedback. We have carefully revised and rewritten the conclusion paragraph in light of your feedback.*

29. References: Some WHO references appear incorrect or non-specific (e.g., Reference 14) & Full reference list should be reviewed once.

- *We appreciate the reviewer's attention to detail. The reference list has been fully reviewed and updated following this suggestion.*

VERSION 2 – REVIEW

**REVIEWER NAME**	Flom, Peter
**REVIEWER AFFILIATION**	Peter Flom Consulting
**REVIEWER CONFLICT OF INTEREST**	No Conflict of Interest
**AI DISCLOSURE**	No, I have not used AI
**DATE REVIEW RETURNED**	09-May-2026

**GENERAL COMMENTS**	The authors have addressed my concerns and I now recommend publication.

**REVIEWER NAME**	Bansal, Utkarsh
**REVIEWER AFFILIATION**	
**REVIEWER CONFLICT OF INTEREST**	No Conflict of Interest
**AI DISCLOSURE**	No, I have not used AI
**DATE REVIEW RETURNED**	19-May-2026

**GENERAL COMMENTS**	All the comments have been adequately addressed, and the necessary corrections have been made in the manuscript.
